# Pharmacokinetic Properties of Adenosine Amine Congener in Cochlear Perilymph after Systemic Administration

**DOI:** 10.1155/2017/8091462

**Published:** 2017-01-18

**Authors:** Hao Chang, Ravindra S. Telang, Sreevalsan Sreebhavan, Malcolm Tingle, Peter R. Thorne, Srdjan M. Vlajkovic

**Affiliations:** ^1^Department of Physiology and Centre for Brain Research, University of Auckland, Private Bag 92019, Auckland 1142, New Zealand; ^2^Auckland Cancer Research Centre, University of Auckland, Private Bag 92019, Auckland 1142, New Zealand; ^3^Department of Pharmacology, University of Auckland, Private Bag 92019, Auckland 1142, New Zealand

## Abstract

Noise-induced hearing loss (NIHL) is a global health problem affecting over 5% of the population worldwide. We have shown previously that acute noise-induced cochlear injury can be ameliorated by administration of drugs acting on adenosine receptors in the inner ear, and a selective A_1_ adenosine receptor agonist adenosine amine congener (ADAC) has emerged as a potentially effective treatment for cochlear injury and resulting hearing loss. This study investigated pharmacokinetic properties of ADAC in rat perilymph after systemic (intravenous) administration using a newly developed liquid chromatography-tandem mass spectrometry detection method. The method was developed and validated in accordance with the USA FDA guidelines including accuracy, precision, specificity, and linearity. Perilymph was sampled from the apical turn of the cochlea to prevent contamination with the cerebrospinal fluid. ADAC was detected in cochlear perilymph within two minutes following intravenous administration and remained in perilymph above its minimal effective concentration for at least two hours. The pharmacokinetic pattern of ADAC was significantly altered by exposure to noise, suggesting transient changes in permeability of the blood-labyrinth barrier and/or cochlear blood flow. This study supports ADAC development as a potential clinical otological treatment for acute sensorineural hearing loss caused by exposure to traumatic noise.

## 1. Introduction

Hearing loss is a growing health problem affecting 360 million people worldwide. Disabling hearing loss refers to hearing loss greater than 40 dB in the better hearing ear in adults and a hearing loss greater than 30 dB in the better hearing ear in children [1]. Noise-induced hearing loss (NIHL) is caused by exposure to high intensity impulse noise or prolonged exposure to moderate or high levels of noise. The lack of hearing protection at work, increasing usage of portable music players, and the frequent exposure to recreational noise are considered to have a significant impact on the incidence of NIHL [2, 3]. Hearing loss (measured as auditory threshold shift) may be temporary (TTS), if a repair mechanism is able to restore the organ of Corti or permanent (PTS) when the sensory hair cells or auditory neurons die. Exposure to continuous or impulse noise can cause mechanical damage and metabolic disruptions in the cochlea of the inner ear [4, 5]. Oxidative stress commences during noise exposure and continues for up to two weeks after noise exposure [6]. Other mechanisms of NIHL include glutamate excitotoxicity, Ca^2+^ overload in sensory hair cells, inflammation, ischemia/reperfusion injury, and cochlear synaptopathy [5, 7].

Currently, prosthetic devices such as hearing aids and cochlear implants are the only treatments for NIHL. Both options are costly, and neither can mitigate cochlear injury. Therefore, there is a strong demand for novel pharmacological or molecular treatments of sensorineural hearing loss (SNHL). Targeting adenosine A_1_ receptors in the cochlea has recently shown promise for the treatment of SNHL [8–12]. Adenosine is an endogenous neuromodulator and a cytoprotective substance released from tissues in response to stress [13, 14]. Adenosine can enhance endogenous antioxidant defenses, increase oxygen supply, improve blood flow, inhibit glutamate release, trigger anti-inflammatory responses, and promote antiapoptotic pathways [14, 15]. Adenosine can also promote angiogenesis, which may be crucial in tissue repair after injury [15, 16].

Previous studies have demonstrated a wide distribution of adenosine receptors in the mammalian cochlea, including chinchillas and rats [18, 19]. All four adenosine receptors (A_1_, A_2A_, A_2B_, and A_3_) are expressed in the rat cochlea, with the strongest immunoexpression of otoprotective A_1_ adenosine receptors (A_1_AR) in sensory hair cells, supporting Deiters' cells and spiral ganglion neurons [19]. Previous studies have shown that noise- and drug-induced hearing loss can be prevented in experimental animals by local or systemic administration of A_1_AR agonists [9, 12, 20, 21]. In 2010, we demonstrated for the first time that the postexposure administration of selective A_1_AR agonists (2-chloro-*N*^6^-cyclopentyladenosine and adenosine amine congener) could ameliorate NIHL in rats [8, 10]. Adenosine amine congener (ADAC) is a synthetic A_1_AR agonist which was designed in a functionalised congener concept [22]. This compound is able to cross the blood brain barrier and is well tolerated by animals after systemic administration. Namely, no cardiovascular side effects (hypotension, hypothermia, and bradycardia) were observed at the dose used for neuroprotection [10, 23–25]. Our study [10] demonstrated that even a single intraperitoneal injection of ADAC 24 hours after traumatic noise exposure partially reversed the hearing loss and reduced sensory hair cell death in rats. A five-day treatment of daily ADAC injections, starting six hours after exposure to noise, was most effective, rescuing approximately 25–30 dB of otherwise permanent hearing loss. The improvement of hearing thresholds was supported by increased survival of sensory hair cells and reduced expression of oxidative stress markers in the cochlea [10]. In a follow-up study, we investigated dose- and time-dependent effects of ADAC in a rat model of NIHL [11]. The dose-response study indicated that ADAC was most effective at 100–200 *μ*g/kg doses. Best effect of the treatment was observed in the first 24 hours after noise exposure, but even delayed treatment after 48 hours provided clinically significant improvement at some hearing frequencies.

However, little is known about the biological fate of ADAC after systemic administration and its pharmacokinetic properties at the target organ (cochlea of the inner ear). Pharmacokinetics describes the movement of a drug through the living organism, including its absorption, distribution, metabolism, and elimination [26]. Understanding the pharmacokinetic properties of ADAC in experimental animals is essential for assessment of its clinical applicability, as it may provide a prediction of ADAC pharmacokinetics in humans [27]. In the present study, we used liquid chromatography-tandem mass spectrometry (LCMS/MS) to establish the pharmacokinetic properties of ADAC in rat perilymph after systemic administration.

## 2. Materials and Methods

### 2.1. Animals

Male Wistar rats sourced from the animal facility at the University of Auckland were used in this study. At the age of 8–10 weeks they were randomly assigned to control or noise-exposed groups (Table 1). The use of animals and experimental procedures were approved by the University of Auckland Animal Ethics Committee and conformed to international guidelines for the ethical use of animals.

### 2.2. Noise Exposure

Rats were exposed to octave-band noise (110 dB SPL, 8–16 kHz bandwidth) for 2 hours to study the effect of acoustic overexposure on ADAC concentrations in perilymph. Noise exposures were carried out in a custom built acoustic chamber (Shelburg Acoustics, Sydney, Australia) equipped with an internal light source and ventilation system. The noise was produced by a noise generator, filtered, amplified, and delivered via two internal speakers suspended from the ceiling of the booth. Two rats per cage were positioned in the centre of the sound chamber directly underneath the suspended speakers. The sound exposure levels within the chamber were measured using a handheld calibrated sound level meter (Rion NL-52, Tokyo, Japan). Control animals were exposed to ambient noise levels in the animal facility (55–65 dB SPL) and kept for two hours in the sound-proof booth without noise exposure.

### 2.3. ADAC Preparation

ADAC (Sigma-Aldrich) was dissolved in 1 M HCl and then in 0.1 M phosphate buffered saline (PBS; pH 7.4) to prepare a 100 *μ*g/mL stock solution. The stock solution was aliquoted and stored at −20°C, and it was later used to prepare calibration standards and injection solutions. When required, ADAC aliquots were heated in a 37°C water bath for 30 min and administered through the femoral vein to animals exposed to traumatic or ambient noise.

### 2.4. ADAC Administration and Perilymph Sampling

Animals were anaesthetised with ketamine (25 mg/kg) and medetomidine (Domitor®, 10 mg/kg) administered intraperitoneally. Once an appropriate depth of anaesthesia was achieved, the skin on the upper medial aspect of the left leg was disinfected with 70% ethanol, followed by a skin incision parallel to the femoral vein. The femoral vein was exposed and the intravenous line inserted for ADAC injection. The rat was supinely positioned under a dissection microscope (Leica Wild M7A) and the head was placed in the head-holder. A heating blanket was used to maintain a rectal temperature at 38°C. The ventral neck region was opened with a large incision, and the salivary and sublingual glands were exposed by separating the fascia and connective tissue and dissected out using thermocautery. The trachea was exposed by bluntly teasing out the sternohyoid muscle, and the endotracheal tube was inserted. The tympanic bulla was exposed by gently teasing away the muscles and connective tissue, and a hole was made in the bulla to expose the cochlea. The cochlea was wiped with tissue paper and dried using compressed air to avoid any seepage and loss of perilymph due to capillary effect. At intervals (1–120 min) after ADAC (100 *μ*g/kg, i.v.) injection, the area corresponding to the scala vestibuli in the apical turn was punctured using a fine needle (~50 *μ*m). A calibrated capillary tube (Harvard Apparatus GC100-10, 1.0 mm OD × 0.58 mm ID) was held over the perforation as soon as the perilymph fluid started oozing out. The total volume of the perilymph collected per cochlea was 2.26 *μ*L. The capillary tubes were calibrated for volume using the ID and length of capillary. To avoid contamination with cerebrospinal fluid (CSF) or endolymph, the volume of 2.26 *μ*L was chosen as lesser than the average rat cochlear perilymph volume (3.02 *μ*L) [28]. The collected perilymph sample was emptied into a vial with 50 *μ*L of saline and transferred to an LC vial with plastic insert (Agilent Technologies). Only one perilymph sample was collected from each cochlea. The samples were then stored at −80°C for LCMS/MS analysis.

### 2.5. LCMS/MS Instrumentation

The LCMS/MS system used in this study was Agilent 1200 series RP-HPLC (Agilent Technologies®, Santa Clara, California) coupled with Agilent 6460 series triple quadrupole mass spectrometer equipped with jet stream electron spray ionisation source (Agilent Technologies). Chromatographic separation was achieved using a Phenomenex® Gemini column (5 *μ*m C18 110 Å, 150 × 3.00 mm) protected with a Phenomenex Gemini C18 guard column (4 × 2 mm ID). The LCMS/MS conditions and instrument settings are summarised in Table 2. Data were acquired and analysed using Agilent MassHunter software. The assay was validated in rat plasma in accordance with the US FDA guidelines on bioanalytical methods for linearity, selectivity/specificity, precision, and accuracy [29]. Selectivity/specificity was examined by collecting blank plasma, homogenised cochlear tissues, and perilymph samples from untreated rats to look for any endogenous peaks that could interfere with the ADAC peak. Accuracy and precision were evaluated by analysing the matrix spike samples at two concentration levels: low QC (1 ng/mL) and high QC (50 ng/mL). Five replicates were examined per QC concentration. Accuracy was calculated by comparing the detected concentration with the true spiked concentration in plasma. Precision was expressed as relative standard deviations (RSD%). Calibration curves obtained in this study included a blank sample, five nonzero samples including the lower limit of quantification (LLOQ). LLOQ was evaluated on the signal to noise ratio of 5 : 1 with precision and accuracy within 20% of the nominal value. Linearity was assessed by preparing calibration curves and plotting the peak area size of ADAC against its concentration.

### 2.6. Data Analysis

Unpaired two-tailed *t*-test was used to compare mean ADAC concentrations in perilymph of noise-exposed and nonexposed animals. The pharmacokinetic (PK) data were analysed by PKSolver [17].

## 3. Results

### 3.1. LCMS/MS Method Development and Validation Procedure

**Figure 1:**
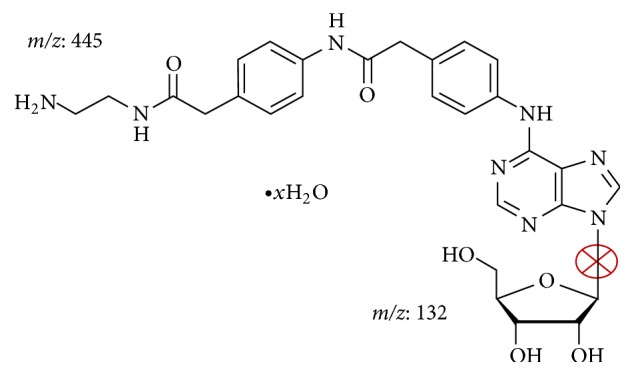
ADAC fragmentation in LCMS/MS. ADAC was fragmented into two daughter ions with* m/z* 445 and 132, respectively. The cross indicates the most likely carbon-nitrogen single bond breakage.

**Figure 2:**
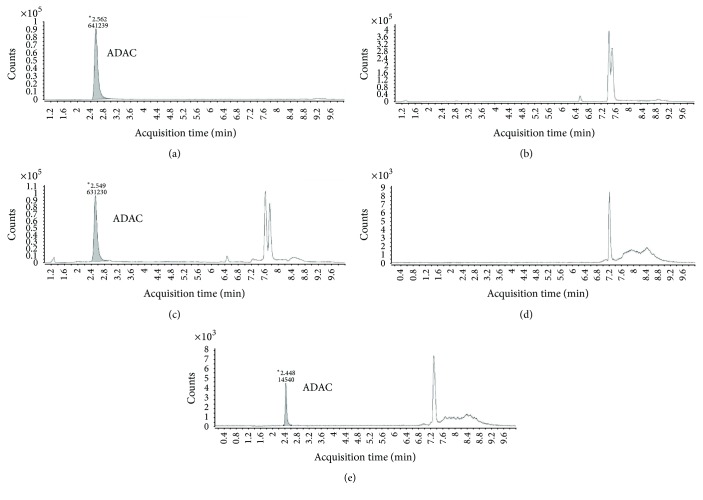
Development of LCMS/MS methods using detection with multiple reaction monitoring (*m/z* 445). The retention time for ADAC is ~2.5 min. (a) ADAC calibration standard (100 ng/mL); (b) cochlear matrix; (c) cochlea spiked with ADAC (10 ng/mL); (d) perilymph matrix; (e) perilymph spiked with ADAC (10 ng/ mL). ^*∗*^Retention time (min).

The LCMS/MS method was developed from the RP-HPLC method for ADAC detection in rat plasma reported in our previous study [11]. During the method development, low collision energy (20 V) was able to shred the ADAC molecule (*m/z* 577) into two daughter ions with* m/z* 445 and 132, respectively. After evaluating the molecular structure of ADAC (Figure 1), the data suggested that the bond breakage occurred at the bond where the side chain joins the ribose sugar. The larger ion (*m/z* 445) presented the stronger signal; hence the final method used multiple reaction monitoring (MRM) for the larger ion only. The run time was 10 min and the retention time ~2.5 min (Figure 2(a)). The homogenised cochlear matrix sample, the cochlear matrix spike sample, the perilymph matrix sample, and the perilymph matrix spike sample are shown in Figures 2(b)–2(e). No endogenous peaks coinciding with the retention time of ADAC were observed in blank plasma (*n* = 5), homogenised rat cochlea (*n* = 6 cochleae from 3 animals), and perilymph samples (*n* = 5) from untreated animals. Accuracy and precision were assessed using QC samples and values are reported in Table 3. Variations for both low and high spikes were less than 15% from the true values. The comparison of LOQ and LLOQ for ADAC using RP-HPLC and LCMS/MS methods is shown in Table 4.

### 3.2. Assessment of ADAC Concentrations in Perilymph by LCMS/MS

**Figure 3:**
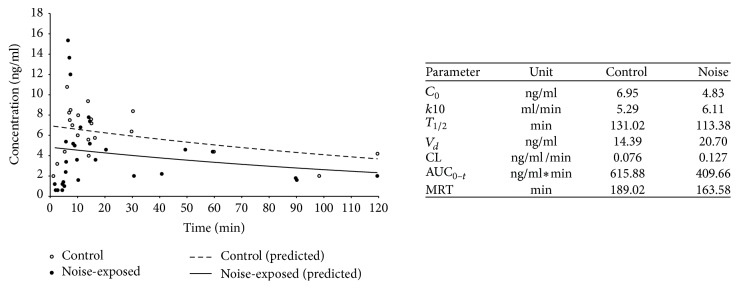
Time-dependent changes in ADAC concentrations in rat cochlear perilymph after intravenous drug administration (100 *μ*g/kg). Time-concentration data were obtained from 22 control cochleae (12 animals) exposed to ambient noise and 31 cochleae (17 animals) exposed to traumatic noise (110 dB SPL, 8–16 kHz, 2 hours). Measured ADAC concentrations (LCMS/MS) were used to plot predicted values. Pharmacokinetic properties were calculated using an Excel plugin PKSolver [17]. *C*_0_, extrapolated drug concentration at time 0; *k*10, elimination constant; *T*_1/2_, drug half-life in perilymph; *V*_*d*_, volume of distribution; CL, drug clearance; AUC_0−*t*_, area under curve (0 − *t*); MRT, mean residence time.

Rats were randomly assigned to a control or noise-exposed group to investigate whether noise exposure had any impact on the permeability of the blood-perilymph barrier and ADAC concentrations in perilymph. After noise exposure, animals were allowed to rest for 1 h before intravenous administration of ADAC, and a single perilymph collection was performed for each cochlea at different intervals over a period of 120 min after ADAC administration.  Figure 3 illustrates changes in ADAC concentrations in perilymph of noise-exposed and nonexposed animals. In control animals exposed to ambient noise, ADAC appeared in the perilymph within 2 min after injection into the femoral vein, peaked at 7 min, followed by drug elimination phase. ADAC was still detectable in the perilymph after 120 min (Figure 3). In noise-exposed animals, ADAC peaked at the same time (*T*max = 7 min) but resulted in higher peak concentration (apparent *C*max = 15.35 ng/mL in noise-exposed versus 10.77 ng/mL in control animals). ADAC concentration dropped quickly in noise-exposed rats after reaching the peak and fluctuated in the first 60 min after administration followed by steady drug elimination phase. The results suggest that ADAC can reach the perilymph promptly after injection into the femoral vein, and its concentration is maintained above MEC for at least 2 h. Comparison of the two experimental conditions revealed higher average drug concentrations in perilymph of the nonexposed cochleae (6.20 ± 2.38; mean ± SD across all time points; *n* = 22) compared to noise-exposed cochleae (4.26 ± 3.80; *n* = 31; *P* = 0.03, unpaired *t*-test). The data were then analysed using PKSolver (an add-in program for pharmacokinetic data analysis in Microsoft Excel) to determine basic PK parameters, including half-life in perilymph, area under curve, and clearance. Drug half-life (*T*_1/2_) and mean residence time (MRT) were shorter in noise-exposed animals (Figure 3). At the same time, area under curve (AUC) was reduced, and the volume of distribution (*V*_*d*_) increased with noise exposure, indicating lower ADAC concentration in perilymph. The comparison of the elimination constant (*k*10) and clearance (CL) indicated faster drug elimination in noise-exposed animals (Figure 3). PK analysis thus suggests that noise exposure affects ADAC absorption, distribution, and elimination in perilymph.

## 4. Discussion

In this study, we have developed and validated the LCMS/MS analytical method to study ADAC pharmacokinetics in cochlear perilymph. ADAC was easily fragmented using low collision energy (20 V) to obtain two daughter ions (*m/z* of 445 and 132) from the parent ion. By reconstructing the two daughter ions back to their original structure, we deduced that the fragmentation took place at the neck in between the side chain and the ribose sugar backbone (Figure 1). Subsequent analysis suggested that the larger ion emitted stronger signal; hence it was selected for the quantitative analysis by multiple reaction monitoring.

Our previous study [11, 30] used UV-Vis detection method to demonstrate a short (5 min) half-life of ADAC in rat plasma after intravenous administration, which was attributed to rapid distribution in tissues. Here, we used a novel LCMS/MS method for ADAC detection in rat perilymph which complies with limits set by US FDA guidelines including accuracy, precision, specificity, and linearity [29]. Most importantly, it was possible to achieve a LLOQ of 0.1 ng/mL, which is three orders of magnitude greater sensitivity compared to the UV-Vis detection method reported previously [11]. As the minimum effective concentration (MEC) for ADAC in rats (0.49 ng/mL) [31] is almost five times higher than LLOQ, our LCMS/MS method was suitable for detection of ADAC at pharmacologically relevant concentrations. The present study demonstrates that the concentration of ADAC in the cochlea after 2 hours is still higher than MEC for this drug, suggesting that the cochlea can maintain the therapeutic concentrations of ADAC for at least 2 h after intravenous administration. We were also able to reduce the i.v. dosage (400 *μ*g/kg) from the study in rat plasma [11] to the standard dose (100 *μ*g/kg) used for the treatment of NIHL [10, 11].

Measuring drug concentration at its proposed site of action provides the most direct evidence of the pharmacokinetics of that drug [32]. To date, there are no pharmacokinetic studies of adenosine A_1_ receptor agonists in cochlear perilymph. The method for ADAC detection in perilymph is a technically demanding procedure as it requires direct perilymph sampling from the cochlear apical turn in anaesthetised animals and a very sensitive analytical method to measure drug concentrations in small volumes (~3 *μ*L). In previous studies, perilymph samples were obtained from the basal turn of the cochlea through an opening in the bony capsule or round window [33, 34]. Such samples are likely to be contaminated with CSF, as scala tympani and the CSF are connected via the cochlear aqueduct near the round window membrane [35]. Therefore, in this study the perilymph was sampled from the apex of the cochlea in order to avoid CSF contamination [32]. After exposing the cochlea, a perforation was made in the apex and only the initial 2.26 *μ*L of perilymph was collected, which is less than estimated perilymph volume in rats (3.02 *μ*L) [28]. Immediate collection and precise measurement of volume reduced the risk of sample contamination by CSF [32].

ADAC was detected in perilymph in the first two minutes after drug injection into the femoral vein. The concentration reached the peak (*C*max) within seven minutes, followed by elimination phase. Traceable amount of ADAC could still be detected at 120 min after administration and the concentrations were above its MEC. Differences in ADAC absorption, distribution, and elimination between the noise-exposed and control animals were likely due to the breach of the blood-labyrinth barrier after noise exposure. The blood-labyrinth barrier is formed by the capillary beds with tight junctions between adjacent endothelial cells and pericytes [36]. The blood-labyrinth barrier in the inner ear regulates the volumes and the composition of the inner ear fluids [37–39]. Numerous studies have suggested that the disruption of this barrier can result in imbalance of local ionic homeostasis [35, 36], reduced drug entry [36], endolymphatic hydrops [40], and hearing loss [41]. A recent study reported the change of blood-labyrinth barrier's permeability after noise exposure [42]. That study identified leaky tight junctions as a possible cause of increased permeability of the blood-labyrinth barrier in the guinea pigs exposed to noise due to decreased cellular expression of Claudin-5 and Occludin [42]. It is possible that noise exposure in our study resulted in leaky tight junctions, leading to a faster ADAC diffusion across the blood-labyrinth barrier. In addition to the compromised blood-labyrinth barrier, noise-induced changes in cochlear blood flow [43] could also affect ADAC pharmacokinetics in perilymph. ADAC fluctuations in cochlear perilymph could also be attributed to cellular uptake and release by nucleoside transporters, as ADAC has a similar structure to adenosine. It has been shown previously that adenosine transporter ENT1 is able to transport tecadenoson, a selective A_1_AR agonist, across the blood brain barrier [44]. Nucleoside transport in the cochlea is sensitive to noise exposure [45], which may result in fluctuations of ADAC concentrations in perilymph.

## 5. Conclusion

Measurements of drug levels in cochlear fluids after local or systemic administration are required to determine their clinical potential for the treatment of inner ear disease. Human pharmacokinetic parameters are often predicted based on animal preclinical pharmacokinetic data [27]; hence pharmacokinetic studies of ADAC using a rat model likely represent a good indicator of clinical concentration-time changes in perilymph. The pharmacokinetic studies revealed that ADAC was able to reach cochlear perilymph rapidly after systemic (intravenous) administration, irrespective of the blood-perilymph barrier. The pharmacokinetic studies also indicated that the minimal effective concentration of ADAC in the cochlea was maintained for at least two hours after intravenous administration, which further underlines the clinical potential of ADAC for the treatment of inner ear injury and hearing loss.

## Figures and Tables

**Table 1:** Experimental groups.

Animal group	Number of cochleae (animals)	ADAC dosage	Administration route	Sample type	Collection period (min)
Control	22 (12)	100 *µ*g/kg	Intravenous	Perilymph	120
Noise exposed	31 (17)	100 *µ*g/kg	Intravenous	Perilymph	120

**Table 2:** The LCMS/MS conditions and instrument settings used to assess ADAC concentrations in rat perilymph.

Min	%B	Flow rate (mL/min)
0	20	0.5
1	20	0.5
5	30	0.5
6	90	0.7
6.5	90	0.7
7.5	20	0.5
10	20	0.5

Mobile Phase A: 4.5 mM ammonium formate pH 7.0.

Mobile Phase B: 100% acetonitrile.

Column: Phenomenex® Gemini 5 *µ*m C18 110 Å, 150 × 3.00 mm S/N: 625787-3.

Injection volume: 20 *µ*L.

Detection wavelength: 254 nm, bandwidth 20 nm.

Source parameters: gas temp: 275°C; gas flow: 10 L/min; nebulizer: 25 psi; sheath gas temp: 350°C; sheath gas flow: 12 L/min. Capillary voltage: 2750 V (positive) and 3500 V (negative); nozzle voltage: 2000 V.

Multiple reaction monitoring (MRM) transitions and compound-dependent parameters: precursor ion (*m/z*) Q1: 577.4; product ion (*m/z*) Q3: 445.4; collision energy: 20 V; fragmentation: 150 V; dwell time: 100 ms.

Run time: 10 min; retention time ~2.5 min.

Thermostat control: 5°C.

**(a):** 

Spiked concentration (*µ*g/mL)	Mean concentration (*µ*g/mL)	Accuracy (%)	Precision (RSD, %)
1.67	1.56	93.41	5.53
16.67	15.22	91.30	12.51

**(b):** 

Spiked concentration (ng/mL)	Mean concentration (ng/mL)	Accuracy (%)	Precision (RSD, %)
1	0.88	88	8.36
50	43.29	86.58	11.95

**(c):** 

Spiked concentration (ng/mL)	Mean concentration (ng/mL)	Accuracy (%)	Precision (RSD, %)
1	0.91	91	10.22
50	44.76	89.52	9.64

**Table 4:** The LOQ and LLOQ for ADAC.

	HPLC (*µ*g/mL)	LCMS/MS (ng/mL)
LOQ	0.05	0.01
LLOQ	0.1	0.1
